# A Rolling Stone Gathers No Moss–The Long Way from Good Intentions to Physical Activity Mediated by Planning, Social Support, and Self-Regulation

**DOI:** 10.3389/fpsyg.2016.01024

**Published:** 2016-07-06

**Authors:** Juliane Paech, Aleksandra Luszczynska, Sonia Lippke

**Affiliations:** ^1^Department of Health Psychology, Focus Area Diversity, Jacobs University BremenBremen, Germany; ^2^Center for Applied Research on Health Behavior and Health, University of Social Sciences and HumanitiesWarsaw, Poland; ^3^Trauma, Health and Hazards Center, University of Colorado, Colorado SpringsColorado Springs, CO, USA

**Keywords:** mediation, HAPA, lifestyle, volition, leisure time activity, SOC

## Abstract

**Background:** Although many people know that an active lifestyle contributes to health they fail to translate their intentions into action. This has been explained by deficits in self-management and resources, such as enabling social support, planning, and self-regulation in the face of barriers. The present study examines the role of perceived social support, planning, and self-regulation in facilitating physical activity.

**Methods:** In a prospective online study, intention was assessed at baseline (Time 1), planning and social support at 4-week follow-up (Time 2), self-regulation and physical activity at 6-month follow-up (Time 3). A path analysis was conducted to shed light on mediating psychological mechanisms contributing to maintenance of physical activity.

**Results:** Perceived support (Time 2), planning (Time 2), and self-regulation (Time 3) mediated the link from intention (Time 1) to physical activity (Time 3); the specific and total indirect effects were significant.

**Conclusions:** Findings suggest that perceived social support, planning, and self-regulation can bridge the intention-behavior gap. Behavior change interventions should target those mechanisms in vulnerable individuals.

## Background

As the proverb suggests, a rolling stone gathers no moss. The same holds true for human beings: Those persons who are physically active on a regular basis do not accumulate as many risk factors with regard to health as inactive people. A *physically active lifestyle* helps to improve muscular and cardio-respiratory fitness as well as bone and functional health, at the same time the risk for coronary heart disease, high blood pressure, stroke, diabetes, and obesity decreases (Lämmle et al., 2014; World Health Organization, WHO, 2014). Physical inactivity, on the other hand, is ranked the fourth leading risk factor for global mortality by the WHO and is assumed to account for approximately one third to one fourth of breast and colon cancers, diabetes, and ischaemic heart disease burden (World Health Organization, WHO, 2014).

Moderate-to-vigorous physical activity results in more pronounced health benefits than physical activity of low intensity (World Health Organization, WHO, 2014). The WHO recommends “at least 150 min of moderate-intensity aerobic physical activity throughout the week or do at least 75 min of vigorous-intensity aerobic physical activity throughout the week or an equivalent combination of moderate- and vigorous-intensity activity” (World Health Organization WHO (2010); p. 26). Although the benefits of physical activity and the risks accompanying inactivity are well-documented in health research, public health studies document that one third of the population worldwide and even 36% of Europeans show insufficient levels of physical activity (World Health Organization, WHO, 2014). To set the stone rolling in terms of behavior change, theory needs to be consulted and applied.

### Theory-driven behavior change

The use of theoretical underpinning should find its way as a standard of good practice into the design and evaluation of studies and interventions in the field of behavior change (Brazil et al., 2005; Medical Research Council, MRC, 2008). In the realm of health promotion research the *Health Action Process Approach* (HAPA; Schwarzer, 2008; Schwarzer et al., 2011) represents a reliable and parsimonious framework to describe and explain health behavior change for a multitude of behaviors and settings. Furthermore, other theoretical frameworks, focusing on the global self-regulatory processes of selective optimization with compensation (SOC; Baltes and Baltes, 1990; Freund and Baltes, 2002) can enrich the understanding of processes involved in behavior change in the face of limited resources.

### Predictors of physical activity

The HAPA differentiates between a motivational and a volitional phase in the health behavior change process. In the first phase, an individual needs to develop the *intention* to change a current health behavior, for example, to become more physically active (Schwarzer, 2008; Schwarzer et al., 2011). This intention needs to be put into action in the volitional phase: The physical activity has to be initiated and maintained (Schwarzer, 2008; Schwarzer et al., 2011). An effective strategy to bridge the intention-behavior gap is *planning*, whereas others resources, such as *social support*, can come into play in the volitional phase as well to underpin the behavior change process (Schwarzer, 2008; Schwarzer et al., 2011).

Recent reviews and meta-analyses indicated consistently that intention is one of the key factors in health behavior. At the same time, many studies show the so-called intention-behavior gap: Although people are highly motivated to adopt or maintain a behavior, they fail to do so because intention cannot sufficiently model behavior change (Bauman et al., 2012; Amireault et al., 2013; Bélanger-Gravel et al., 2013; Rhodes and Bruijn, 2013). Despite strong intentions many individuals fail to translate their goals into action (Schwarzer, 2008). Intention formation is the main focus in the motivational phase and it represents the starting point for behavior initiation (Schwarzer, 2008; Schwarzer et al., 2011). Goal intentions need to be automatized and linked with specific cues to action. This automation can bridge the gap between intention and behavior by shielding intentions from situational obstacles. After intentions have been formed, action needs to be prepared in the volitional phase where post-intentional processes substantiate the subsequent steps of behavior change (Schwarzer, 2008; Schwarzer et al., 2011).

Other social-cognitive determinants are of considerable importance to bridge the gap between intention and behavior. Volitional strategies set the stage for the automation of intentions and help to initiate and maintain health behaviors, such as physical activity, and planning has empirically been found to be a mediator of the intention-behavior relationship (Gollwitzer and Sheeran, 2006; Schwarzer, 2008; Bélanger-Gravel et al., 2013; Hagger and Luszczynska, 2014; Mistry et al., 2015). A combination of *action and coping plans* is the most effective approach with regard to health behavior interventions (Bélanger-Gravel et al., 2013). Some self-regulatory challenges with regard to a physically active lifestyle can be overcome by determining the when, where, and how of intended activities (action plans) and also what to do in the face of barriers (coping plans). Other volitional variables are social support and self-regulation, which can further increase the likelihood that a high intention is translated into behavior.

*Social support* can give an impetus to maintain health behavior (Bauman et al., 2012; Amireault et al., 2013; Tay et al., 2013). When individuals are motivated to change they will consider the available resources that can help them in the initiation of a new behavior. The question is *how* they can put their intention into action. Research showed that received social support may explain the adoption of a healthy diet over and above other cognitions (Scholz et al., 2013). In turn, perceived social support was shown to enhance global self-regulation and operate in concert with other variables (Anderson et al., 2010; Anderson-Bill et al., 2011). The appraisal of available social support becomes particularly meaningful after the process of intention formation since social support is a proximal predictor of behavior and can make up for internal deficiency such as setbacks with self-efficacy (Schwarzer, 2008; Schwarzer et al., 2011). Thus, perceived social support may be seen as another linking mechanism in the intention-behavior relationship.

*Global self-regulation* processes may represent another crucial link in the intention-behavior chain working in orchestration with social support and planning (Anderson et al., 2010; Reuter et al., 2010; Anderson-Bill et al., 2011). The model of selection, optimization, and compensation (SOC; Baltes and Baltes, 1990; Freund and Baltes, 2002) describes the organization of behavior in the light of limited individual resources which enables an adaptive development (Baltes and Baltes, 1990). The concept originates in lifespan theory: Age-related decline and loss are met with coping strategies that focus on achievable goals (selection), maximize gains within the given framework (optimization), and fulfill ambitions by alternative approaches (compensation; Baltes and Baltes, 1990). Even beyond aging theory, SOC strategies can be fruitfully applied in the setting and accomplishment of health goals, for example by prioritizing health goals that cannot be achieved simultaneously. When applied in the context of health behavior change, the SOC model provides an extensive repertoire of self-regulatory strategies which aid initiation and maintenance of a physically active lifestyle. Identifying relevant SOC strategies may enrich the understanding of complex behavioral mechanisms, such as physical activity, not only from a loss-based perspective of life cycle but also from a global life management view (Son et al., 2009; Reuter et al., 2010).

### Aims of the study

Aim of the present study is to examine linking mechanisms of the intention-behavior relationship and to analyze the interplay between intention, planning, perceived social support, and global self-regulation with regard to moderate-to-vigorous physical activity in an overall model. Prior research has investigated selected parts of the proposed overall model (e.g., Son et al., 2009; Reuter et al., 2010; Anderson-Bill et al., 2011) but the complex interplay of intention, planning, social support, and SOC strategies was not fully addressed before. The current study seeks to expand the existing body of research by an integrative approach that takes the central determinants of behavior into consideration, in particular in the domain of physical activity.

**Figure 1:**
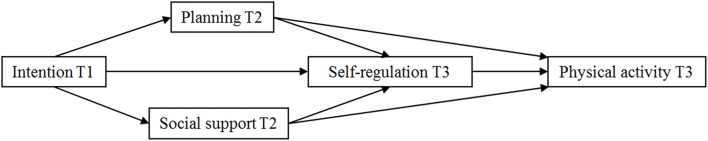
**Hypothesized mediation model**.

The primary objective was to model the complex intention-behavior relationship and identify to which extent planning, perceived social support (measured at Time 2), and global self-regulation strategies (measured at Time 3) mediate the link between intention to be physically active (measured at Time 1) and moderate-to-vigorous physical activity (measured at Time 3; see Figure 1).

## Methods

### Participants and procedure

The present data come from a German online longitudinal study which was conducted using the software dynQuest (Rademacher and Lippke, 2007). The study was advertised in the local press and on German websites. Potential respondents had to give informed consent to receive the link to the self-administered questionnaire. The start page was entered and informed consent given by 2207 individuals. The initial response rate was reduced to a number of 1752 participants who provided their e-mail addresses for follow-up assessments. Those who answered at least 50% of the questions at T1 were considered as enrolled participants. At Time 1 (T1) *N* = 991 participated in the survey. Respondents were invited to follow-up assessments at 4 weeks (Time 2, T2; *n* = 742) and at 6 months (Time 3, T3; *n* = 463) later. There were no significant differences found between those who answered more than half of the questions and those who responded to less (all *p*s > 0.05).

The study was conducted in consideration of the Helsinki Declaration. All participants received the same questionnaire and no clinical or experimental treatment.

### Measures

At T1 intentions, baseline physical activity, age and gender were assessed. The T2 questionnaire measured planning and perceived social support. At T3 self-regulation and physical activity were assessed. The employed measures have been validated before and can be regarded as valid and reliable research instruments. All psychological variables were measured using 4-point Likert scales ranging from 1 (totally disagree) to 4 (totally agree).

**Intentions to become more physically active** were measured as suggested by Nigg (2005), matching different levels of physical activity intensity. This measure was validated in earlier studies (Nigg, 2005). An index of two items was used corresponding to the behavior measurement: Strenuous and moderate physical activities. A sample from the questionnaire was “I intend to perform the following activities at least 5 days per week for 30 min strenuous (rapid heartbeats, sweating) physical activities.” The item intercorrelation was *r* = 0.17 reflecting the heterogeneity of the aggregated constructs.

**Perceived social support from family or friends** for regular physical activity was assessed by two items (Jackson et al., 2011). Participants were asked how they perceive their environment and responded, for example, to the following item: “My family helps me to be physically active.” The item intercorrelation was *r* = 0.39.

**Planning** was assessed by measuring action and coping planning with three items each (Ziegelmann and Lippke, 2007). Action plans were characterized by the when, where and how of physical activity; the sample item was “I have already planned when and how often I will be physically active.” The coping planning measure assessed plans for physical activity in the face of barriers, e.g., “I have already precisely planned what to do if something intervenes.” Item intercorrelations ranged from *r* = 0.42 to 0.90. Both subscales were combined into a general planning index. The reliability coefficient for the index was Cronbach's α = 0.90.

**Self-regulation** was measured with an adapted version of the SOC strategies questionnaire (Freund and Baltes, 2002). This adapted version of the SOC questionnaire assesses the use of self-regulation strategy in the domain of healthy lifestyle and has been validated by Reuter et al. (2010). The scale consisted of four items, reliability in the present sample was good (Cronbach's α = 0.82). A sample item was “When it is getting more difficult to lead a healthy lifestyle I only strive for my most important health goal.”

**Physical activity** was assessed by a modified version of the Godin Leisure-Time Exercise Questionnaire (GLTEQ; Godin and Shephard, 1985; Plotnikoff et al., 2007). The self-report measure asked participants to provide information on the average number of sessions per week and average duration in minutes per week of their leisure-time physical activity. An index of moderate (not exhausting, light perspiration) and strenuous activities (rapid heartbeats, sweating) was built to reflect the range of intensity levels in physical activity as recommended by the World Health Organization WHO (2010). A sum score was built from the responses (product of frequency and duration) for each intensity level.

### Data analysis

Manifest path analysis using Mplus 6.1 was applied to test the hypothesized model (see Figure 1). Missing data (<11% on all variables) were treated by Full Information Maximum Likelihood estimation to make use of all available information in the model. Predictors were mean-centered. Baseline behavior, gender (1 = female, 2 = male) and age were included as covariates. Bias-corrected bootstrapping (5000 samples) was employed to aid data non-normality and to estimate indirect effects (Preacher et al., 2007).

## Results

### Preliminary analysis

Participants in the final analyzed sample were 16–78 years of age (*M* = 37.83, *SD* = 12.27). The sample consisted of 72.8% women; 55% of the participants were married or living with a partner; 77.7% had completed senior high school; and 53.0% held a university degree.

Dropout analyses revealed no differences between those who completed the study and those who dropped out after T1 (all *p*s > 0.05) except for the age of the completers who were slightly younger than those who did not respond after T1 [*t*_(949)_ = −3.01, *p* = 0.003].

Means, standard deviations and intercorrelations of all variables can be found in Table 1. All social-cognitive variables were significantly positively interrelated. The highest correlation occurred between planning (T2) and self-regulation (T3), which may be due to their volitional character. But as they share only 17% of variance and show differential patterns with the other variables construct validity could be assumed for each of the two constructs and they were included as distinct variables in the analyses. Gender was positively associated with age and physical activity, and negatively with intention T1. Age showed significant negative associations with planning T2 and physical activity T1.

**Table 1:** **Means, standard deviations, ranges, and intercorrelations of study variables**.

	***M***	***SD***	**Range**	**2**	**3**	**4**	**5**	**6**	**7**	**8**
1. Gender	–	–	1/2	0.24^**^	−0.08^*^	0.02	−0.04	−0.05	0.07^*^	−0.01
2. Age	37.83	12.27	16-78		0.04	−0.03	−0.09^*^	0.07	−0.11^**^	−0.03
3. Intention T1	2.56	0.78	1-4			0.13^**^	0.26^**^	0.20^**^	0.20^**^	0.13^**^
4. Social support T2	2.12	0.85	1-4				0.27^**^	0.22^**^	0.15^**^	0.10^**^
5. Planning T2	2.73	0.81	1-4					0.41^**^	0.21^**^	0.19^**^
6. Self-regulation T3	2.44	0.66	1-4						0.16^**^	0.23^**^
7. Physical activity T1	151.24	157.69	0-630							0.33^**^
8. Physical activity T3	116.18	139.06	0-630							

** *p < 0.01*,

* *p < 0.05, Gender: 1 = female, 2 = male; T1, Time 1; T2, Time 2, 4-week follow-up; T3, Time 3, 6-month follow-up*.

### Mediation analysis

We used multiple mediation analysis, assuming parallel mediators (planning and perceived social support) which operated in a sequence with self-regulation, to test the model. In particular, the model assumed the indirect (via planning at T2 and perceived social support at T2, respectively, and self-regulation at T3) effects of intention to be physically active at baseline on physical activity 6 months later. The model was controlled for baseline behavior, gender and age. Model-data fit was very good, χ^2^ (1, *N* = 901) = 1.19, *p* = 0.28; RMSEA = 0.01 (90% CI [0.00; 0.09]); CFI = 1.00, TLI = 0.99, SRMR = 0.01.

**Figure 2:**
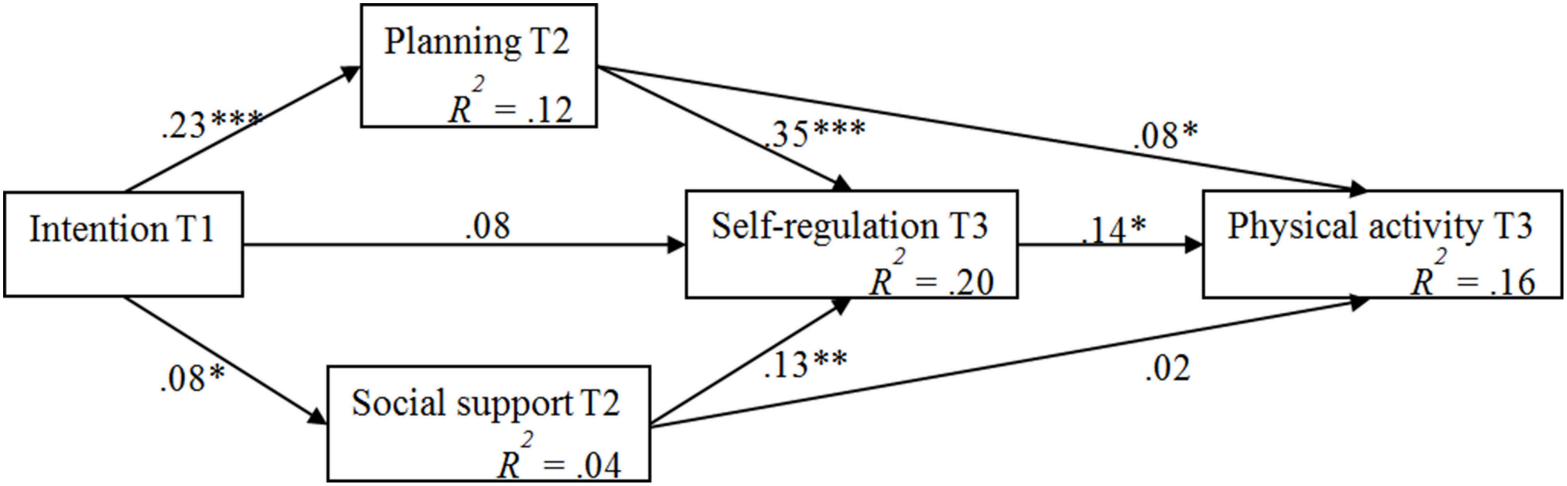
**Mediation model with standardized coefficients (controlled for gender, age, baseline behavior)**. T1, Time 1; T2, Time 2, 4-week follow-up; T3, Time 3, 6-month follow-up. ^***^p < 0.001; ^**^p < 0.01; ^*^p < 0.05.

Figure 2 and Table 2 show the standardized and unstandardized path coefficients along with the explained variance in each variable. The results of mediation analysis corroborate the assumed associations between the social-cognitive variables: Intention at T1 predicted planning (T2) and perceived social support (T2) which, in turn, were significantly associated with self-regulation (T3). Planning (T2) and self-regulation (T3) predicted physical activity (T3). Furthermore, baseline physical activity was significantly associated with planning (T2), perceived social support (T2), and physical activity (T3); age was also significantly related to planning at T2 (all *p*s < 0.05, see Table 2).

**Table 2:** **Model results with unstandardized path coefficients**.

	***B***	***SE***	***p***
**PLANNING T2**			
Intention T1	0.25	0.04	< 0.01
Gender	< −0.01	0.06	0.98
Age	0.01	< 0.01	0.03
Physical activity T1	< 0.01	< 0.01	< 0.01
**SOCIAL SUPPORT T2**			
Intention T1	0.09	0.05	0.05^a^
Gender	0.05	0.06	0.37
Age	< −0.01	< 0.01	0.13
Physical activity T1	< 0.01	< 0.01	< 0.01
**SELF-REGULATION T3**			
Planning T2	0.28	0.04	< 0.01
Social Support T2	0.10	0.04	< 0.01
Intention T1	0.07	0.03	0.06
Gender	−0.01	0.05	0.82
Age	< 0.01	< 0.01	0.26
Physical activity T1	< 0.01	< 0.01	0.37
**PHYSICAL ACTIVITY T3**			
Self-regulation T3	28.98	11.28	0.01
Planning T2	14.03	6.35	0.03
Social support T2	3.88	7.15	0.59
Gender	−1.01	7.67	0.90
Age	−0.17	0.36	0.63
Physical activity T1	0.28	0.04	< 0.01

a *p = 0.046; T1, Time 1; T2, Time 2, 4-week follow-up; T3, Time 3, 6-month follow-up*.

All estimated indirect effects were significant. Intention T1 had specific indirect effects on physical activity T3 through planning T2 and self-regulation T3, *B* = 1.98, BC 95% CI [0.59; 3.92], and via perceived social support T2 and self-regulation T3, *B* = 0.27, BC 95% CI [0.03; 0.99]. The total indirect effect was significant, *B* = 7.92, BC 95% CI [4.32; 12.68].

## Discussion

The study aimed to broaden the understanding of linkages between the intention to be physically active and moderate-to-vigorous physical activity at 6-month follow-up. As hypothesized, planning and perceived social support, respectively, as well as global self-regulation mediated the relationship between intention and physical activity measured six months later. Prior findings showed the effects of planning, social support or self-regulation strategies operating directly or as single mediators (e.g., Reuter et al., 2010; Anderson-Bill et al., 2011). The present study provides novel evidence and explains how the three social-cognitive variables may operate jointly, forming a specific sequence.

High intentions enhanced global self-regulation and subsequently physical activity 6 months later when individuals had planned their activities and perceived their social environment as highly supportive in the meantime. High levels of intention resulted in more planning and more perceived social support which both in turn were associated with higher levels of global self-regulation and thus led to higher levels of physical activity. The indirect effects were significant confirming the hypothesized mediation model.

The results provide support for the crucial role of planning in adoption and maintenance of healthy behavior (cf. Schwarzer, 2008). Planning may be defined as a strategy fostering further conscious and effortful self-regulation processes (Hagger and Luszczynska, 2014). In line with this assumption, we found that planning prompted the use of conscious and effortful self-regulatory strategies, which in turn predicted physical activity. The global self-regulation strategy is primarily concerned with health in general and therefore aiming at a higher level of achievement. The use of a paramount health-focused self-regulatory strategy may drive the transfer of concrete plans into behavior, reinforcing initiation, and maintenance of physical activity from an operational perspective (Reuter et al., 2010). Self-regulatory strategies can come into effect on a specific, state-like level, when action plans and coping plans guide behavior, and on a more global, trait-like level, when pursuing, coordinating, and attaining higher-level goals, such as health and well-being which can only be achieved via concrete intermediate steps and outcomes. Individual resources can be effectively replenished with social resources, as we have outlined in our research model. The complementary needs of individuals in the process of health behavior change can be adequately met in an integrated resource-oriented approach (Schwarzer, 2008; Anderson-Bill et al., 2011; Schwarzer et al., 2011). Thus, research on determinants of health behavior change should be based on integrative models that reflect the different levels of individual goal pursuit.

The indirect effect of intention on physical activity via social support and self-regulation may seem weaker than the indirect effect of intention on physical activity via planning and self-regulation. Furthermore, the explained variance in social support (see Figure 2, *R*^2^ = 0.04) was relatively low indicating that other predictors should be included besides intention, e.g., characteristics of the perceived supporters such as self-efficacy (Ayotte et al., 2013). Additionally, the sources of support could be further differentiated to understand their individual contribution to physical activity in the long run. Future studies may also further investigate the differential effects of perceived and received social support (cf. Scholz et al., 2013).

### Limitations of the study and further directions

Some limitations have to be considered in the present study and overcome in future research. The longitudinal design of the study could be expanded to longer follow-ups as prior research has shown that physical activity levels may decrease after 6 months and only stabilize in the following years (Reuter et al., 2009). Despite the theoretical underpinning and the time-lag design only limited causal inferences can be drawn from the results. Future studies should apply the experimental manipulation to gain more insight into cause and effect relationships. Another important aspect is the refinement of the applied measures to strengthen the basis of valuation and the reliability of the results. In particular the scales assessing intentions and perceived social support consisted of only two items each and aggregated relatively heterogeneous aspects of the operationalized constructs. The reliability coefficients and intercorrelations turned out to be rather low indicating the heterogeneity of the indices and therefore limiting the significance of the results. The current instruments need to be developed further to provide a solid basis for research. Furthermore, the outcome of physical activity was based on self-reported data. Subjective measures may be prone to response or recall bias (Prince et al., 2008). This should be taken into account when interpreting the results.

While the study shed some light on how individuals can put their intentions into action the question of moderators remains untouched. Those linking mechanisms might work for certain subgroups particularly well while others might need something else to become physically active on a regular basis. Future studies should include specific moderators of the intention-behavior relationship such as intention stability, conscientiousness, or anticipated regret (e.g., Rhodes and Dickau, 2013).

### Outlook

To understand processes of the uptake and maintenance of complex behaviors such as moderate-to-vigorous physical activity—-in other words to initiate and maintain that a rolling stone gathers no moss—-several social-cognitive variables have to be taken into consideration. Intention, planning, and social support have been derived as important predictors from the theoretical framework of the HAPA (Schwarzer, 2008; Schwarzer et al., 2011). The construct of global self-regulation (Baltes and Baltes, 1990; Freund and Baltes, 2002) replenished the strategic repertoire of highly motivated individuals on their way to a physically active lifestyle and the explanatory model in our study.

## Authors contributions

All authors conceived the study. JP performed the statistical analysis and drafted the manuscript. SL designed the study and participated in revisions of the manuscript. AL contributed to the revisions of the manuscript. All authors read and approved the final manuscript.

## Funding

The contribution of AL and the preparation of this manuscript were supported by grant 2014/15/B/HS6/00923 the National Science Center, Poland.

### Conflict of interest statement

The authors declare that the research was conducted in the absence of any commercial or financial relationships that could be construed as a potential conflict of interest.
